# The clinical significance of S100B, APP, and CHI3L1 in autoimmune GFAP astrocytopathy

**DOI:** 10.3389/fneur.2026.1801196

**Published:** 2026-04-08

**Authors:** Lu Liu, Boya Ma, Jiahui Peng, Ning Li, Yinghui Zhang, Qiang Liu, Juan Yang, Li Zhao, Yi Li, Yanbai Wang, Xiao Yang

**Affiliations:** Department of Neurology, General Hospital of Ningxia Medical University, Yinchuan, China

**Keywords:** autoimmune GFAP astrocytopathy, cerebrospinal fluid biomarkers, correlation, pathophysiology, serum biomarkers

## Abstract

**Background:**

Autoimmune glial fibrillary acidic protein astrocytopathy (A-GFAP-A) is a recently defined immune-mediated disorder of the central nervous system (CNS). At present, diagnosis relies primarily on the detection of GFAP-IgG in cerebrospinal fluid (CSF); however, the limited specificity of this biomarker restricts its clinical utility. This study aimed to investigate the expression profiles and clinical relevance of additional protein biomarkers in A-GFAP-A.

**Methods:**

A total of 19 patients with A-GFAP-A, 28 patients with neuromyelitis optica spectrum disorder (NMOSD), 12 patients with non-inflammatory neurological diseases (NINDC), and 12 healthy controls (HC) were enrolled. Serum and CSF levels of S100 calcium-binding protein B (S100B), amyloid precursor protein (APP), and chitinase-3-like protein 1 (CHI3L1) were quantitatively measured using enzyme-linked immunosorbent assay (ELISA). Disease severity was assessed using the Expanded Disability Status Scale (EDSS). Correlations between biomarker levels and clinical parameters were analyzed to evaluate their diagnostic and pathophysiological significance.

**Results:**

Serum S100B levels were significantly higher in the A-GFAP-A group than in the NMOSD group (*p* < 0.01), whereas CSF S100B levels were lower than those in NMOSD but remained higher than in the NINDC group (*p* < 0.05). Serum APP concentrations were markedly elevated in A-GFAP-A compared with both NMOSD and HC (*p* < 0.05 and *p* < 0.001, respectively). CSF APP levels did not differ significantly between A-GFAP-A and NMOSD but were significantly higher than in NINDC (*p* < 0.001). CSF CHI3L1 was specifically and significantly increased in A-GFAP-A (*p* < 0.01) and differed markedly from NINDC (*p* < 0.01). Correlation analysis revealed a significant positive correlation between serum S100B and EDSS score (r = 0.642, *p* = 0.013). GFAP-IgG titers were strongly correlated with both serum and CSF APP levels (serum: r = −0.676, *p* = 0.008; CSF: r = 0.826, *p* = 0.001).

**Conclusion:**

Serum S100B and APP, as well as CSF APP and CHI3L1, show potential as auxiliary diagnostic biomarkers for A-GFAP-A. These biomarkers may contribute to differential diagnosis and provide insights into disease mechanisms, thereby supporting more precise clinical management.

## 1 Introduction

Autoimmune glial fibrillary acidic protein astrocytopathy (A-GFAP-A) is a rare and recently recognized type of central nervous system (CNS) autoimmune disease that is currently understood mainly through case series and retrospective studies. Since the first report of a GFAP-IgG–positive case of meningoencephalomyelitis in 2016, the disease spectrum has continuously expanded to include multiple clinical phenotypes such as meningitis, meningoencephalitis, and myelitis (1, 2). Cerebrospinal fluid (CSF) GFAP-IgG is currently considered a key diagnostic marker for A-GFAP-A, however, due to the rarity of the disease and the extensive overlap in clinical, pathological, and molecular features with other neuroimmunological disorders, its differential diagnosis in clinical practice remains highly challenging.

This overlap is particularly pronounced at the level of antibody expression and broader molecular biomarkers. Existing literature indicates that GFAP-IgG is not absolutely specific for A-GFAP-A and is frequently detected as a coexisting antibody in other central nervous system immune-mediated diseases, including neuromyelitis optica spectrum disorder (NMOSD), myelin oligodendrocyte glycoprotein antibody-associated disease (MOGAD), and autoimmune encephalitis (3, 4) and may also be present in non-neuroimmunological conditions (5). Moreover, with respect to conventional cerebrospinal fluid inflammatory biomarkers, A-GFAP-A often exhibits high similarity to the aforementioned demyelinating diseases, lacking sufficient discriminatory specificity. More strikingly, some patients presenting with typical meningoencephalomyelitis accompanied by autonomic dysfunction test negative for GFAP-IgG, yet their responses to immunotherapy do not differ significantly from those of antibody-positive patients (3, 4). Pathophysiological studies further reveal that the core pathological feature of A-GFAP-A is CD8^+^ T cell–mediated astrocytic injury, accompanied by perivascular lymphocytic infiltration and microglial activation (6). However, the downstream cascade following astrocyte lysis, including blood–brain barrier disruption, axonal transport impairment, and neuroinflammation, has not yet been clearly elucidated (7, 8). Of particular note, GFAP-IgG may represent a secondary phenomenon, as it cannot directly bind to the intracellular GFAP antigen and is more likely an immunological byproduct of T cell–mediated cytotoxicity following astrocytic destruction (9). The complex overlap of antibodies, the nonspecific nature of conventional molecular markers, and the limitations in current mechanistic understanding greatly constrain the accuracy of single-antibody–based diagnostic strategies, underscoring the urgent need to identify more specific and complementary biomarkers.

Based on this background, the present study focuses on biomarkers reflecting astrocytic injury and its secondary pathological consequences, including the blood–brain barrier integrity marker S100 calcium-binding protein B (S100B), the axonal transport impairment marker amyloid precursor protein (APP), and the neuroinflammatory marker chitinase-3-like protein 1 (CHI3L1). S100B is a calcium-binding protein widely regarded as a marker of astrocytic injury, and its release is typically associated with cell membrane disruption or astrocyte activation (10). As the precursor of amyloid proteins, APP is closely associated with metabolic abnormalities in neurodegenerative diseases, however, its role in neuroimmunological disorders has not yet been fully elucidated (11). In immune-mediated diseases such as multiple sclerosis, APP is highly expressed in active demyelinating plaques and localized to T lymphocytes, foamy macrophages, activated microglia, and reactive astrocytes, with expression levels correlating with lesion progression, suggesting that APP may serve as a sensitive marker of disease activity (12). CHI3L1 is primarily secreted by reactive astrocytes, and its expression under neuroinflammatory conditions can be induced by proinflammatory stimuli such as interleukin-1β or tumor necrosis factor-*α*, possibly via activation of the nuclear factor-κB signaling pathway (13). By measuring the levels of these biomarkers in the serum and cerebrospinal fluid of patients with A-GFAP-A and analyzing their correlations with clinical parameters, this study aims to identify biological indicators that may assist in diagnosis and disease assessment.

In addition, given that NMOSD also primarily targets astrocytes and shares overlapping clinical manifestations and vulnerable anatomical sites, it was selected as a disease control; comparison with NMOSD, whose pathophysiological mechanisms are relatively well defined (14), may help further clarify the disease characteristics of A-GFAP-A and provide data for identifying biomarkers that distinguish the two conditions (9).

## 2 Methods

### 2.1 Study population

The experimental group consisted of patients with acute-phase A-GFAP-A who were hospitalized in the Department of Neurology at General Hospital of Ningxia Medical University from January 2022 to December 2024 (specific inclusion criteria are detailed below).

A three-group control system was established:

- A disease control group of patients with acute-phase NMOSD [meeting the 2015 International Consensus Diagnostic Criteria for NMOSD (15)]. and positive for aquaporin-4 antibodies;
- Age-and sex-matched healthy controls (HC);
- Patients with non-inflammatory neurological diseases (NINDC) as CSF-negative controls.

All patients were diagnosed by three physicians experienced in neuroimmunological disorders; for diagnostically challenging cases, multidisciplinary expert consultations were held. Patients meeting the inclusion criteria were preliminarily enrolled, biological specimens were collected, and clinical data were recorded. Written informed consent was obtained from all participants. The study protocol was reviewed and approved by the Ethics Committee of Ningxia Medical University General Hospital (Approval No: KYLL-2025-2405).

### 2.2 Inclusion criteria

#### 2.2.1 A-GFAP-A patients

- Acute or subacute onset, with clinical manifestations including fever, headache, involuntary movements, myelitis, ataxia, psychiatric or behavioral abnormalities, autonomic dysfunction, and other meningoencephalomyelitic symptoms;
- CSF positivity for GFAP-IgG (cell-based assay);
- Exclusion of alternative diagnoses.

#### 2.2.2 NMOSD patients

- Fulfillment of the 2015 International Consensus Diagnostic Criteria for NMOSD (15);
- All enrolled NMOSD patients tested positive for aquaporin-4 antibodies (AQP4-IgG) and were in either the first or a relapsing acute attack.

#### 2.2.3 NINDC patients

Patients undergoing lumbar puncture due to non-inflammatory neurological conditions (e.g., headache or seizure, but ultimately excluding intracranial infection and other inflammatory neurological disorders). Their CSF samples were used for comparative analysis with A-GFAP-A and NMOSD groups to exclude confounding effects of non-inflammatory factors on biomarker levels.

### 2.3 Exclusion criteria

- Incomplete clinical data;
- Refusal to provide informed consent.

### 2.4 Sample exclusion criteria

- Samples with unclear or missing information;
- Contaminated samples;
- Samples not meeting preservation requirements, including those with gross hemolysis (serum appearing dark red), severe lipemia (serum appearing milky and viscous), or visible precipitation upon inspection.

### 2.5 Research methods

#### 2.5.1 Collection of biological specimens

##### 2.5.1.1 Blood samples

From pre-enrolled patients, peripheral venous blood was collected into EDTA anticoagulant tubes and serum separation tubes before the initiation of immunotherapy. Blood samples were also collected from healthy controls. All blood samples were drawn from the cubital vein in the early morning after an overnight fast, with approximately 5 mL collected per subject. Samples were transported to the laboratory within 4 h for centrifugation (3,000 rpm, 10 min). Subsequently, 2 mL of serum and 2 mL of plasma were aliquoted into sterile 2 mL cryovials. After anonymized coding and labeling, specimens were stored at −80 °C for subsequent analysis.

##### 2.5.1.2 CSF samples

CSF samples were obtained via routine lumbar puncture from pre-enrolled patients before the initiation of immunotherapy. Under strict aseptic technique, 2 mL of CSF was collected into sterile 2 mL cryovials. After anonymized coding and labeling, samples were stored at −80 °C until analysis.

#### 2.5.2 Detection of biological markers

The concentrations of S100B, APP, and CHI3L1 in serum and CSF were quantified using enzyme-linked immunosorbent assay (ELISA) based on the double-antibody sandwich principle. All procedures were strictly followed according to the manufacturer’s protocols. To minimize subjective bias, ELISA operators were blinded to group allocation and only had access to anonymized sample codes. Group assignments were disclosed only after completion of testing, data entry, and data lock for statistical analysis.

#### 2.5.3 Kits and catalog numbers

- S100B ELISA Kit (Lianke Bio, A116440613);
- APP ELISA Kit (Yuer Sheng Bio, L240922699);
- CHI3L1 ELISA Kit (Yuer Sheng Bio, L240919526).

#### 2.5.4 Statistical analysis

All analyses were performed using SPSS 26.0 and GraphPad Prism software. The statistical analyses in this study were performed using SPSS 26.0 and GraphPad Prism software. Measurement data are presented as mean ± standard deviation. The choice of statistical methods was based on the distribution characteristics of the data: for data that passed normality tests, differences between two groups were assessed using the independent samples t-test. Comparisons among multiple groups under the assumption of homogeneity of variance were conducted using one-way analysis of variance (ANOVA); if the homogeneity of variance assumption was violated, the Kruskal-Wallis H non-parametric test was employed. For variables that did not follow a normal distribution, as determined by normality tests, data are described using the median (interquartile range). The Mann–Whitney Utest was used for comparisons between two groups, and the Kruskal-Wallis H test was applied for comparisons across multiple groups. Categorical variables are summarized as frequencies, and differences between groups were evaluated using the Chi-square test or Fisher’s exact probability method, as appropriate. Correlations between variables were analyzed using Spearman’s rank correlation. The significance threshold was set at a two-tailed *p* < 0.05.

## 3 Results

### 3.1 Expression levels of S100B, APP, and CHI3L1 in serum and CSF

**Figure 1:**
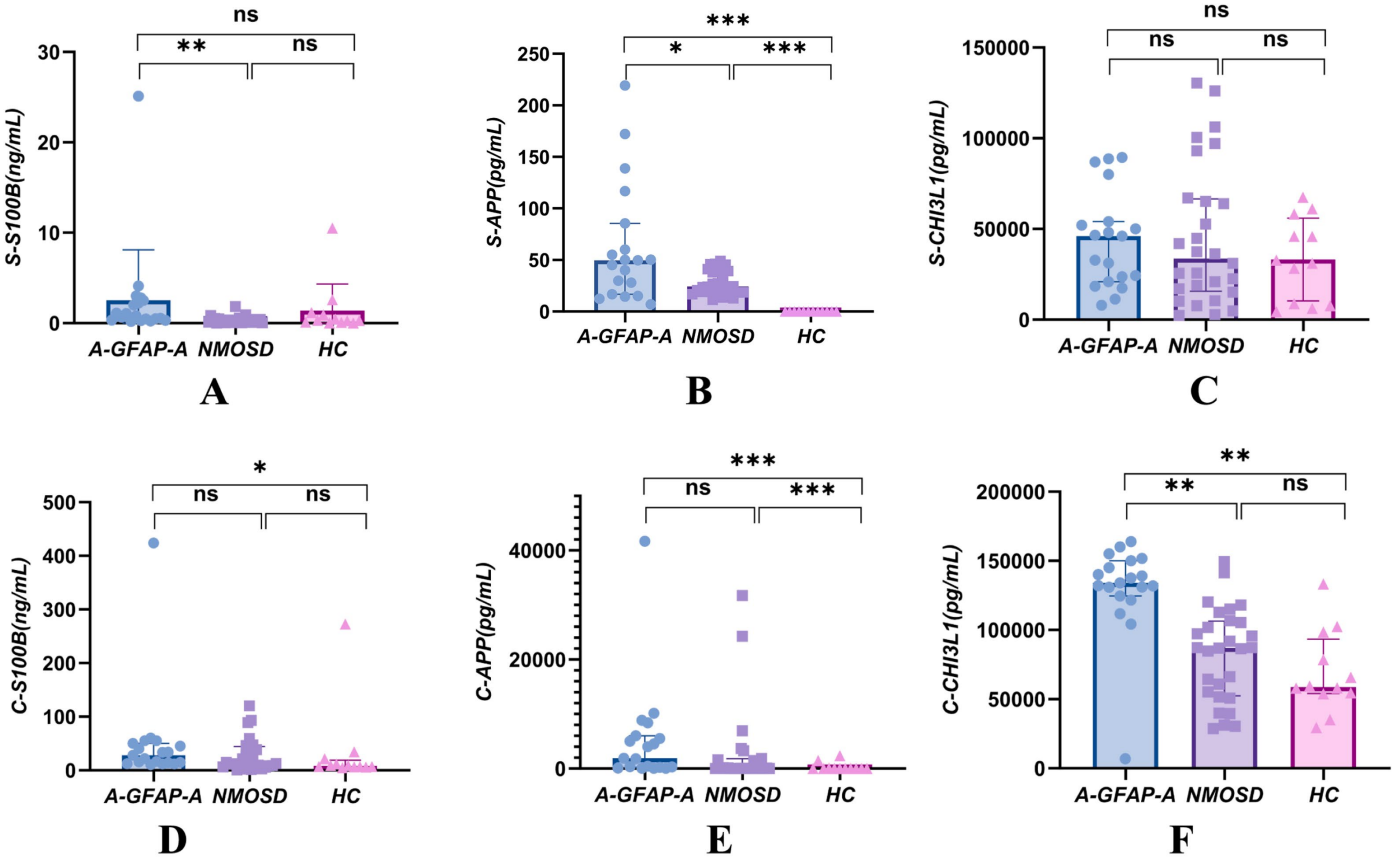
Concentrations of S100B, APP, and CHI3L1 in serum and CSF across study groups. **(A)** Serum S100B, **(B)** serum APP, **(C)** serum CHI3L1, **(D)** CSF S100B, **(E)** CSF APP, **(F)** CSF CHI3L1. Data are presented as mean ± SD. (A–A), Autoimmune GFAP astrocytopathy; NMOSD, neuromyelitis optica spectrum disorder; HC, healthy controls; NINDC, non-inflammatory neurological disease controls (*p < 0.05, **p < 0.01, **p < 0.001; ns, not significant).

We compared the levels of S100B, APP, and CHI3L1 in serum and CSF across the A-GFAP-A, NMOSD, NINDC, and HC groups. Significant inter-group differences were observed for multiple biomarkers, as summarized in Figure 1.

In serum, S100B levels were significantly elevated in the A-GFAP-A group compared to the NMOSD group (*p* < 0.01; Figure 1A). Similarly, serum APP levels were markedly higher in A-GFAP-A than in both the NMOSD and HC groups (*p* < 0.05 and *p* < 0.001, respectively; Figure 1B). In contrast, no statistically significant differences were found in serum CHI3L1 levels among the A-GFAP-A, NMOSD, and HC groups (Figure 1C).

**Figure 2:**
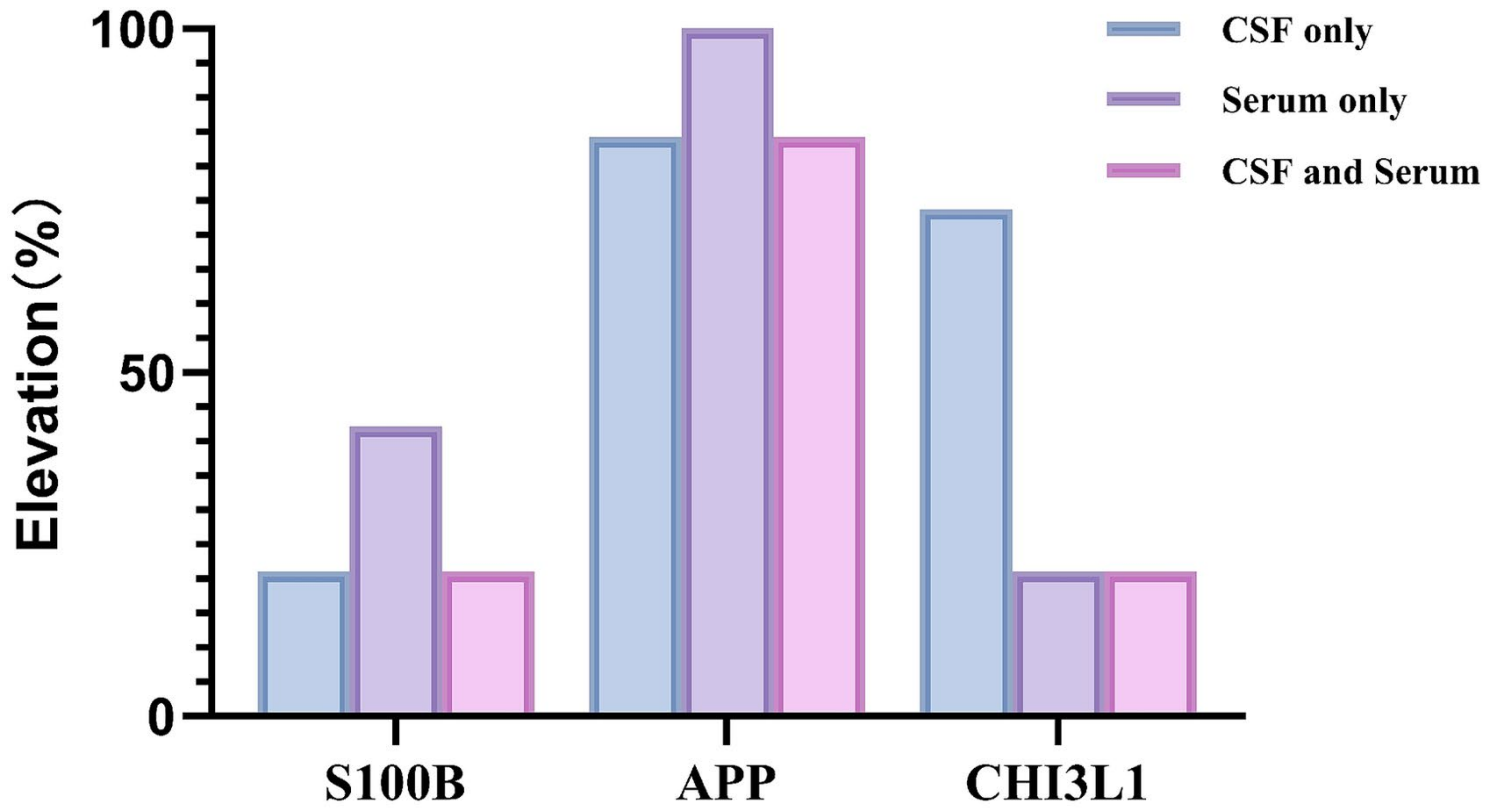
In paired serum and CSF samples, elevations of S100B and APP were predominantly observed in serum, whereas CHI3L1 elevation was more concentrated in CSF. This distribution pattern indicates distinct analyte specificity (elevation defined as values exceeding the mean + 2 SD of healthy controls).

**Figure 3:**
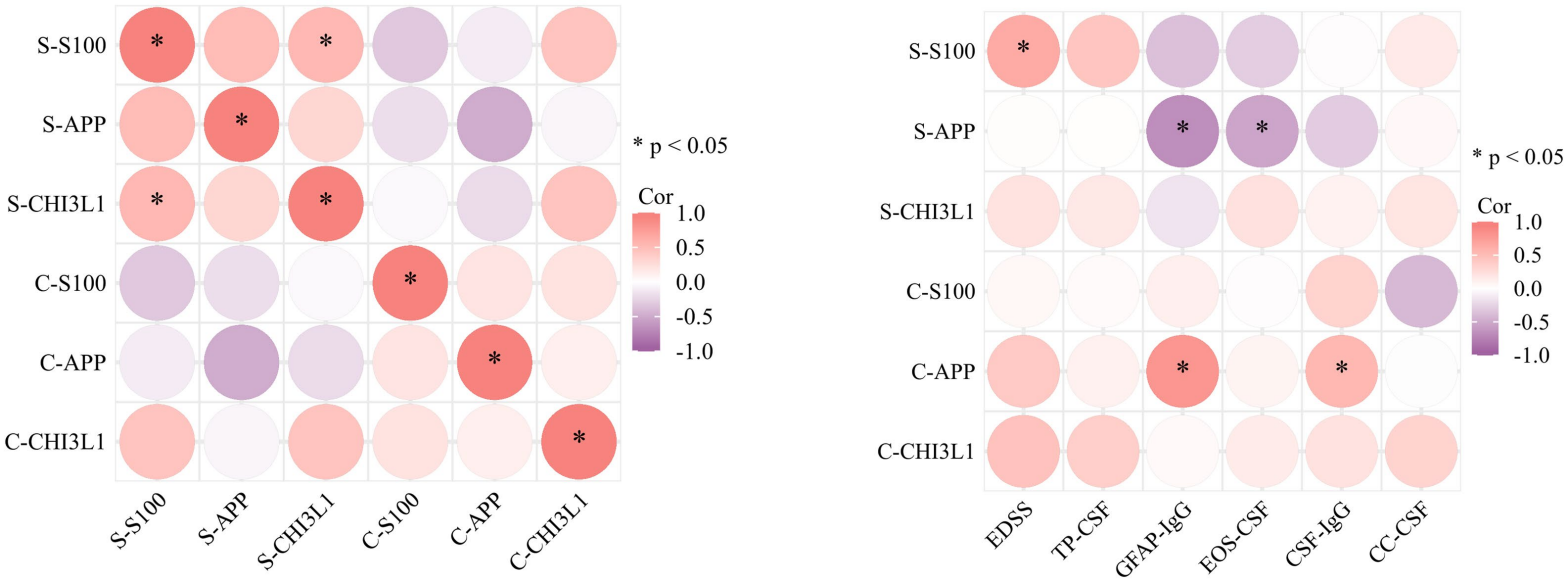
Correlation heatmap of biomarkers and clinical parameters in the A-GFAP-A cohort. Left panel: Pairwise correlations among serum (S-) and cerebrospinal fluid (C-) biomarkers. Right panel: Correlations between biomarkers and clinical indices (TP-CSF: total protein; CC-CSF: cell count; EOS-CSF: eosinophil percentage; EDSS: Expanded Disability Status Scale). Color intensity and size of the circles represent the strength of the Spearman’s correlation coefficient, as indicated in the legend.

In CSF, the levels of all three biomarkers were altered in the A-GFAP-A group. CSF S100B levels in A-GFAP-A were lower than those in NMOSD but remained significantly higher than in the NINDC group (*p* < 0.05; Figure 1D). For CSF APP, no significant difference was observed between A-GFAP-A and NMOSD; however, both disease groups exhibited markedly higher levels than the NINDC group (*p* < 0.001; Figure 1E). Most notably, CSF CHI3L1 was highly elevated in the A-GFAP-A group, showing significant differences compared to both the NMOSD and NINDC groups (*p* < 0.01 for both; Figures 1F, 2, 3).

### 3.2 Correlation between biomarkers and clinical features in A-GFAP-A

**Figure 4:**
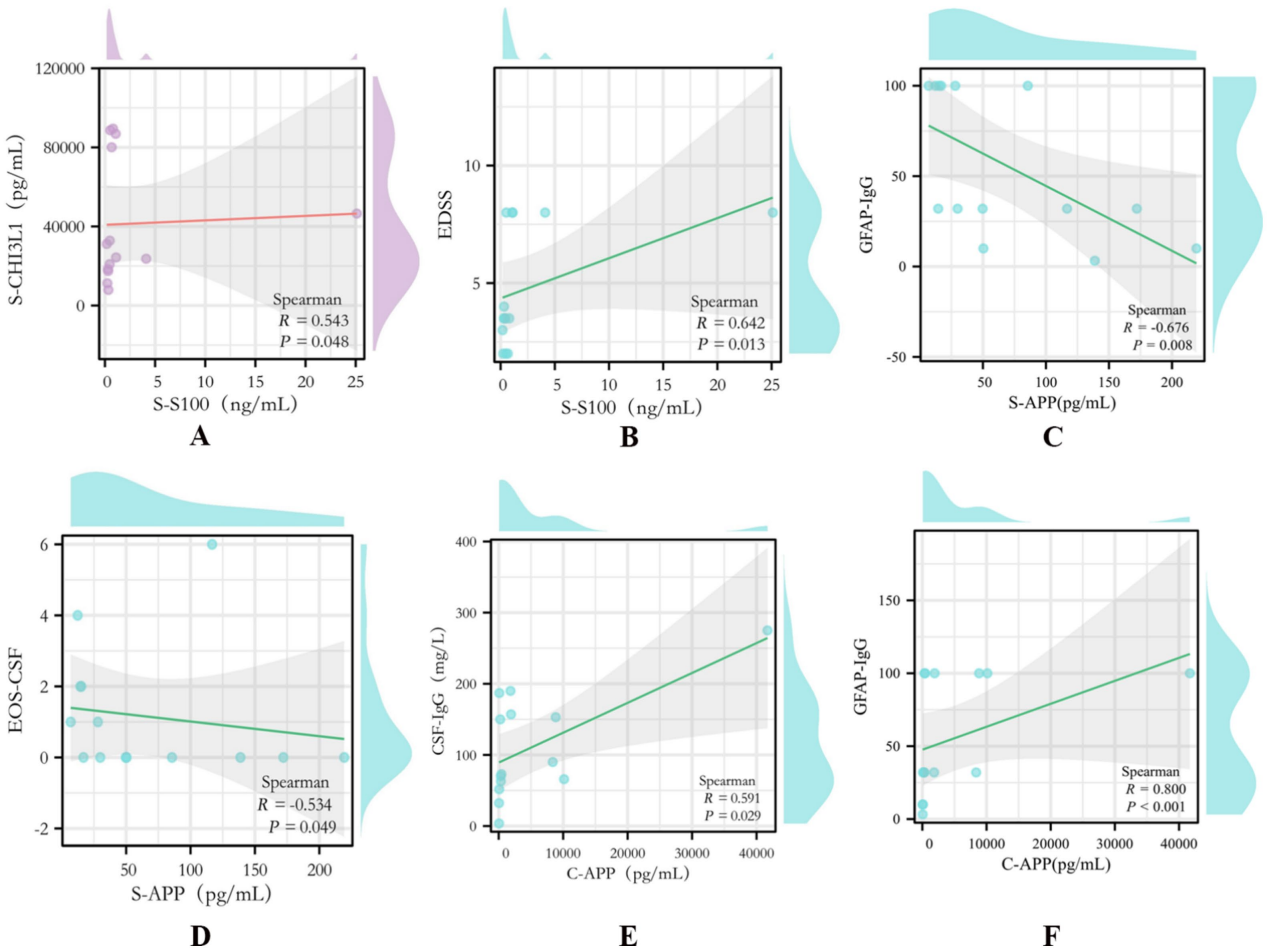
Scatter plots of significant correlations between biomarkers and clinical parameters in A-GFAP-A. **(A)** Serum S100B vs. serum CHI3L1 (*p* = 0.048, *R* = 0.543). **(B)** Serum S100B vs. EDSS score (*p* = 0.013, *R* = 0.642). **(C)** Serum APP vs. GFAP-IgG titer (*p* = 0.008, *R* = −0.676). **(D)** Serum APP vs. CSF eosinophil percentage (EOS-CSF) (*p* = 0.049, *R* = 0.534). **(E)** CSF APP vs. CSF IgG (*p* = 0.029, *R* = 0.591). **(F)** CSF APP vs. GFAP-IgG titer (*p* < 0.001, *R* 0.800). The solid line represents the line of best fit from linear regression analysis.

To investigate the clinical relevance of the assessed biomarkers, we analyzed their correlations with key demographic, laboratory, and clinical severity parameters in the A-GFAP-A cohort (Table 1). A comprehensive correlation matrix of all measured variables is presented in Figure 4.

**Table 1:** Baseline clinical and demographic characteristics of the study cohort with A-GFAP-A.

Patient ID	Age	Sex	TP-CSF (g/L)	CSF-IgG (mg/L)	CSF Cell Count	GFAP-IgG (CSF)	EDSS (peak)	Eosinophils (CSF)	Main clinical presentation	Tumor
1	27	Male	4.47	3.7	172.0	1:3.2	8	0	Meningoencephalomyelitis	No
2	49	Male	0.98	70.0	5.0	1:32	3.5	0	Myelitis	No
3	53	Male	1.45	73.0	185.0	1:100	8	0	Meningoencephalomyelitis	No
4	19	Male	2.14	157.0	267.0	1:32	8	0	Meningitis	No
5	59	Male	0.96	190.0	85.0	1:100	8	1	Meningoencephalitis	No
6	48	Male	1.30	51.0	185.0	1:10	2	0	Meningitis	No
7	71	Male	1.19	90.0	45.0	1:32	8	0	Meningoencephalomyelitis	No
8	54	Male	2.37	153.0	162.0	1:100	4	1	Myelitis	No
9	41	Male	3.18	150.0	152.0	1:32	3.5	2	Meningitis	No
10	37	Female	2.65	187.0	375.0	1:32	2	6	Meningitis	No
11	55	Male	0.80	63.0	432.0	1:100	3	2	Meningitis	No
12	65	Male	3.51	275.0	122.0	1:100	3.5	0	Meningitis	No
13	33	Male	1.27	66.0	180.0	1:100	3.5	4	Meningitis	No
14	65	Male	0.56	28.5	10.0	1:10	2	0	Meningoencephalitis	No
15	38	Female	2.3	35	387	1:32	4	0	Meningoencephalitis	No
16	62	Male	1.83	157	200	1:3.2	8	0	Myelitis	No
17	56	Male	1.85	170	22	1:10	4	0	Meningoencephalomyelitis	No
18	64	Male	0.91	93.2	40	1:10	2	0	Meningitis	No
19	55	Male	2.72	222	140	1:32	8	4	Meningoencephalomyelitis	No

The EDSS score represents the maximum disability rating assessed during the acute admission phase. As the Expanded Disability Status Scale (EDSS) was originally designed. For multiple sclerosis (MS), its applicability to A-GFAP-A may be limited.

Serum S100B level demonstrated a significant positive correlation with the disease severity score EDSS (r = 0.642, *p* = 0.013; Figure 4B) and was also positively correlated with serum CHI3L1 concentration (R = 0.543, *p* = 0.048; Figure 4A).

Both serum and CSF APP levels showed strong correlations with the GFAP-IgG antibody titer, albeit in opposite directions. Serum APP was negatively correlated with GFAP-IgG titer (R = −0.676, *p* = 0.008; Figure 4C), whereas CSF APP was positively correlated (R = 0.800, *p* <0.001; Figure 4F). Furthermore, serum APP showed a positive correlation with the percentage of CSF eosinophils (R = 0.534, *p* = 0.049; Figure 4D), and CSF APP was positively correlated with CSF IgG level (R = 0.591, *p* = 0.029; Figure 4E).

**Figure 5:**
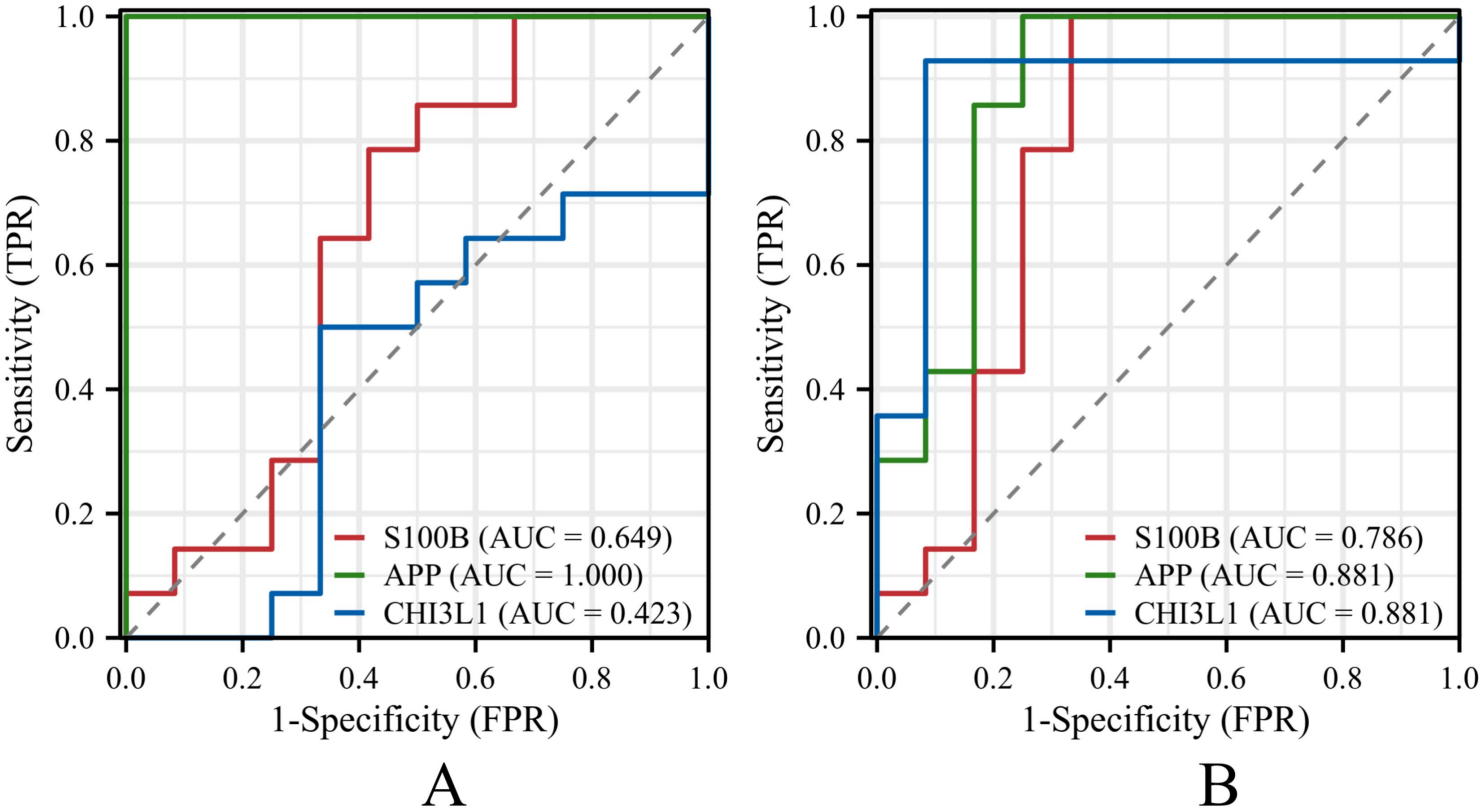
**(A)** ROC curve analysis of three serum biomarkers (S100B, APP, CHI3L1). The ROC curves demonstrate the discriminative performance of S100B (AUC = 0.649), APP (AUC = 1.000), and CHI3L1 (AUC = 0.423). **(B)** ROC curve analysis of three CSF biomarkers (S100B, APP, CHI3L1), showing AUC values of 0.786, 0.881, and 0.881, respectively. Since serum APP levels in healthy controls were below the assay detection limit, while those in the A-GFAP-A group were consistently measurable with no overlap between groups, the serum APP yielded an AUC value of 1.000.

To further assess the diagnostic performance of the investigated biomarkers, receiver operating characteristic (ROC) analyses were performed (see Figure 5).

## 4 Discussion

This study systematically evaluated the expression levels and clinical associations of S100B, APP, and CHI3L1 in A-GFAP-A, revealing that serum S100B and APP, as well as CSF APP and CHI3L1, exhibit potential value as auxiliary diagnostic biomarkers for A-GFAP-A.

We found that serum S100B concentrations were significantly higher in patients with A-GFAP-A than in those with NMOSD and healthy controls (Figure 1A), suggesting a potential involvement of S100B in the pathophysiology of A-GFAP-A. Previous studies have shown that under pathological conditions, high concentrations of S100B can activate proinflammatory signaling pathways through binding to the receptor for advanced glycation end products (RAGE), thereby amplifying neuroinflammation (16–18). We hypothesize that astrocytic damage in A-GFAP-A leads to massive release of S100B into the circulation, where elevated peripheral S100B not only reflects tissue injury but may also exacerbate blood–brain barrier disruption through a positive feedback loop mediated by the S100B–RAGE axis. To further explore the clinical relevance of this biomarker, correlation analyses were performed, demonstrating a significant positive correlation between serum S100B levels and EDSS scores (R = 0.642, *p* = 0.013; Figure 4B). This finding indicates that elevated serum S100B is closely associated with more severe neurological disability, further supporting its potential utility as a biomarker for assessing disease severity in A-GFAP-A.

In contrast to serum findings, CSF S100B concentrations in the A-GFAP-A group were slightly lower than those in the NMOSD group and showed a relatively narrow distribution (Figure 1D). This apparent discrepancy may be related to the kinetic properties of the S100B protein (19). Under conditions of mild brain injury with selective blood–brain barrier opening, S100B may preferentially diffuse unidirectionally from CSF into the bloodstream, and differences in sampling time windows may capture a phase characterized by declining CSF levels but persistently elevated serum concentrations (20, 21). In addition, the relatively high clearance rate of CSF through arachnoid granulations further limits the residence time of S100B in CSF, accelerating its decline in concentration (22). Consequently, S100B released from these lesions may more readily enter the systemic circulation through local microvascular leakage rather than diffusing directly into the CSF. By contrast, deep lesions in the spinal cord or optic nerve in NMOSD may result in sustained leakage of S100B into the CSF (23, 24).

Regarding axonal injury and immune activation, the significant elevation of serum APP in the A-GFAP-A group also demonstrates important discriminatory value (Figure 1B). Previous studies have demonstrated that APP-derived products, such as amyloid-*β*, can activate inflammasomes including NLRP3, promoting proinflammatory cytokine release and exacerbating neuroinflammation (25–27). This is consistent with our observation of significantly elevated serum APP in A-GFAP-A, suggesting that APP may serve as a marker of peripheral immune activation. CSF APP levels did not differ significantly between A-GFAP-A and NMOSD (Figure 1E) but were markedly higher than those in non-inflammatory controls, these findings suggest that elevated CSF APP may represent a common response to astrocytic injury (28). However, the strong correlation between CSF APP and GFAP-IgG levels (R = 0.826; Figure 3E) further supports the involvement of APP in autoimmune pathways. APP may amplify astrocytic injury by modulating microglial phenotypic polarization or inflammatory signaling pathways. Therefore, upregulation of APP not only reflects astrocytic dysfunction but may also contribute to disease progression in A-GFAP-A by promoting a self-perpetuating neuroinflammatory cycle.

In addition, both previous reports and our data indicate an increased proportion of eosinophils in the CSF of patients with A-GFAP-A (29, 30), This increase is significantly greater than that observed in NMOSD and MOGAD, However, the clinical significance and underlying immunopathological mechanisms remain unclear. In this study, CSF eosinophil counts showed a moderate positive correlation with serum APP levels (R = 0.534, *p* = 0.049; Figure 4D). This observation is concordant with pathological findings reported by Tanner et al. in cases of amyloid-*β*–related angiitis (ABRA) (31). Brain biopsy in these cases revealed extensive Aβ deposition within vessel walls, frequently accompanied by eosinophilic infiltration. Whether elevated APP in A-GFAP-A promotes abnormal Aβ deposition in cerebral microvessels and subsequently triggers CNS immune responses characterized by eosinophilic infiltration warrants further investigation.

With respect to the neuroinflammatory marker CHI3L1, CSF concentrations were significantly higher in the A-GFAP-A group than in other groups (Figure 1F), Levels reached the order of 10^6^ pg./mL, whereas serum CHI3L1 concentrations did not differ significantly among groups (Figure 1C), This pattern highlights its central role in localized CNS inflammation. Previous studies suggest that CHI3L1 may contribute to the maintenance and expansion of the neuroinflammatory microenvironment by inhibiting oligodendrocyte progenitor proliferation and differentiation or by promoting astrocyte migration (32). Taken together, the markedly elevated CSF CHI3L1 levels in A-GFAP-A, in contrast to unchanged serum levels, underscore its high tissue specificity as a biomarker of localized CNS inflammation. These findings not only indicate a central role of CHI3L1 in sustaining the neuroinflammatory milieu of A-GFAP-A but also further support its candidacy as a CSF biomarker.

Despite these findings, several limitations of the present study should be acknowledged. First, A-GFAP-A is a rare disease with highly heterogeneous clinical manifestations and no universally accepted diagnostic criteria, making case identification and enrollment challenging. As a result, the relatively small sample size may limit statistical power and the robustness of biomarker evaluation. Second, the cross-sectional design precluded longitudinal follow-up of patients. This limitation restricts a comprehensive interpretation of the findings and their potential clinical implications. Given the rarity of A-GFAP-A and uncertainty regarding the timing of clinical diagnosis, systematic longitudinal follow-up remains challenging in real-world settings. Consequently, dynamic monitoring of biomarkers across different disease stages and before and after immunotherapy was not available. This limitation prevents evaluation of the true utility of S100B, APP, and CHI3L1 in predicting relapse, monitoring treatment response, and guiding individualized management. Future multicenter, large-scale prospective longitudinal cohort studies are urgently needed to validate the dynamic trajectories of these biomarkers throughout the disease course of A-GFAP-A and to clarify their potential for clinical translation.

## Data Availability

The original contributions presented in the study are included in the article/supplementary material, further inquiries can be directed to the corresponding author.
